# Opioid Prescribing by US Surgeons, 2016-2022

**DOI:** 10.1001/jamanetworkopen.2023.46426

**Published:** 2023-12-07

**Authors:** Jason Zhang, Jennifer F. Waljee, Thuy D. Nguyen, Amy S. Bohnert, Chad M. Brummett, Mark C. Bicket, Kao-Ping Chua

**Affiliations:** 1 Department of Pediatrics, Susan B. Meister Child Health Evaluation and Research Center, University of Michigan Medical School, Ann Arbor; 2 Section of Plastic Surgery, Department of Surgery, University of Michigan Medical School, Ann Arbor; 3 Opioid Prescribing Engagement Network, Institute for Healthcare Policy and Innovation, University of Michigan Medical School, Ann Arbor; 4 Department of Health Management and Policy, University of Michigan School of Public Health, Ann Arbor; 5 Department of Anesthesiology, University of Michigan Medical School, Ann Arbor; 6 Opioid Research Institute, University of Michigan, Ann Arbor

## Abstract

This cross-sectional study investigates the rate and dosing of opioid prescriptions among US surgeons from 2016 to 2022.

## Introduction

US surgeons prescribe opioids more frequently than surgeons elsewhere.^1^ These prescriptions often exceed patient need, increasing diversion risk.^2^ In response, policies and practice guidelines centered on opioid prescribing for acute pain have been implemented.^3,4^

Timely national data on opioid prescribing by surgeons are important to inform ongoing stewardship initiatives. However, the most recent data on this prescribing come from 2019.^5^ We addressed this gap using national data from 2016 to 2022.

## Methods

This cross-sectional study used data from the IQVIA Longitudinal Prescription Database, which captures 92% of prescriptions dispensed in US retail pharmacies. Because data are deidentified, the Institutional Review Board of the University of Michigan exempted this study from review. This study follows the STROBE reporting guideline for cross-sectional studies.

We included opioid prescriptions from surgeons dispensed between 2016 and 2022. We excluded prescriptions for non-US patients and those with invalid or missing dosing data. Outcomes were the monthly surgical opioid dispensing rate (dispensed opioid prescriptions from surgeons per 100 000 people), monthly mean total morphine milligram equivalents (MMEs) per prescription (a standardized measure of prescription size), and monthly total MMEs per 100 000 people.

To assess trend changes, we fitted joinpoint regression models. We calculated unadjusted changes in outcomes between January 2016 and December 2022 by specialty. Analyses used Joinpoint Trend Analysis Software version 5.0.2 (National Cancer Institute) and 2-sided hypothesis tests with α = .05 (eMethods in Supplement 1).

## Results

Of 143 256 192 opioid prescriptions, 2 669 942 prescriptions (1.9%) were excluded. The remaining 140 586 250 opioid prescriptions were for 67 922 137 patients (40 927 459 females [60.3%]; mean [SD] age at first fill during the study period, 47.5 [19.0] years).

During January 2016 to December 2022, the monthly surgical opioid dispensing rate decreased from 661.2 to 426.0 prescriptions per 100 000 people (35.6%). This rate decreased 0.89% (95% CI, −0.96% to −0.81%) per month during January 2016 to January 2020, declined sharply and rebounded during February to July 2020, and declined 0.45% (95% CI, −0.61% to −0.29%) per month from August 2020 onward (Figure, A).

**Figure.**
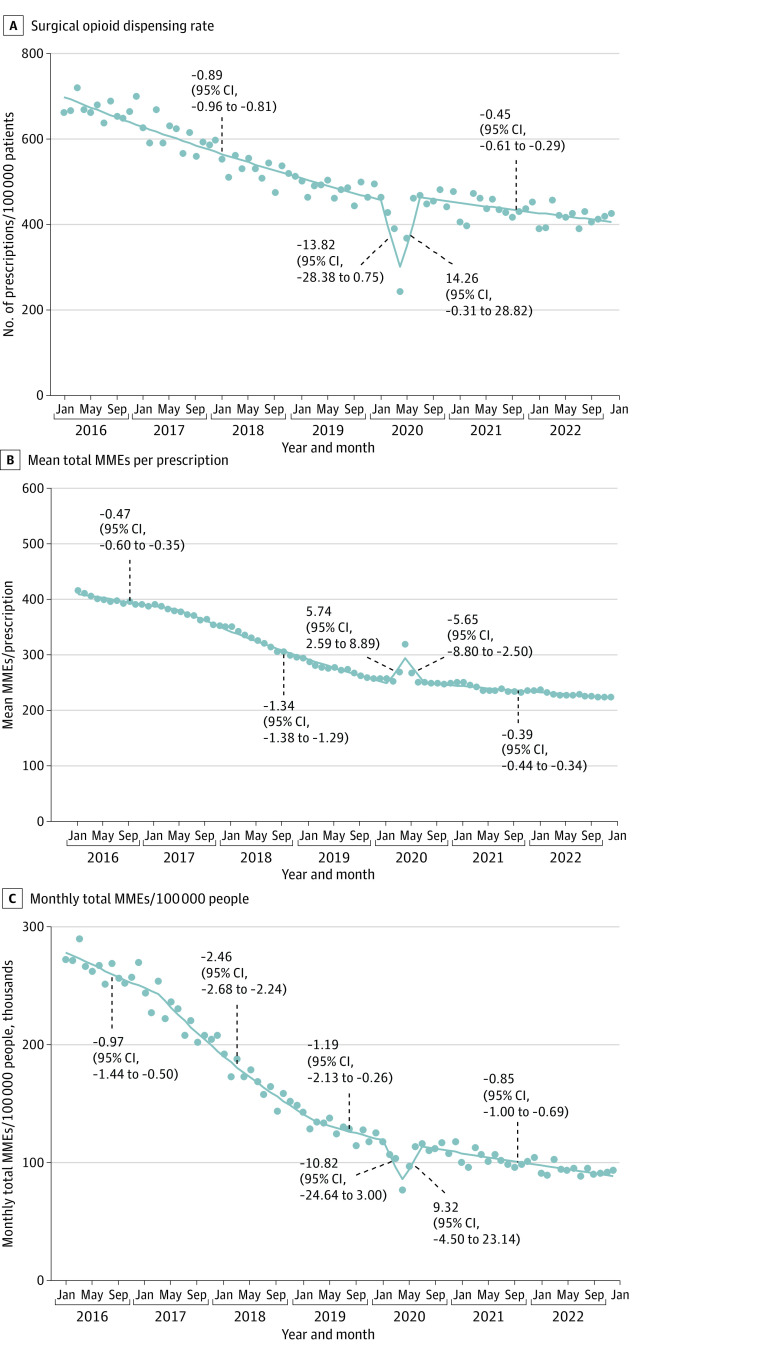
Rate and Dosing of Dispensed Opioid Prescriptions From US Surgeons, 2016-2022 The surgical opioid dispensing rate is defined as the number of dispensed opioid prescriptions from surgeons per 100 000 people. For each segment, the monthly percentage change (95% CI) is displayed. Surgeons were defined as physicians specializing in cardiothoracic surgery, general surgery (including colorectal and transplant surgery), neurosurgery, obstetrics and gynecology, ophthalmology, orthopedic surgery (including hand surgery), otolaryngology, pediatric surgery, plastic surgery, thoracic surgery, urology, or vascular surgery. MME indicates morphine milligram equivalent.

Monthly mean total MMEs per prescription decreased from 414.0 to 222.0 prescriptions (approximately 44 pills containing 5 mg hydrocodone) during January 2016 to December 2022 (46.4%). This quantity decreased 0.47% (95% CI, −0.60% to −0.35%) per month during January 2016 to May 2017 and 1.34% (95% CI, −1.38% to −1.29%) during May 2017 to January 2020. After an increase and decrease during February to July 2020, this quantity declined 0.39% (95% CI, −0.44% to −0.34%) per month from August 2020 onward (Figure, B).

During January 2016 to December 2022, monthly total MMEs per 100 000 people decreased from 273 746 to 94 548 MMEs (65.5%). Similar to other outcomes, this quantity decreased less rapidly after mid-2020 compared with before 2020 (Figure, C).

The Table shows changes in outcomes between January 2016 and December 2022 by specialty. For orthopedic surgery, the specialty accounting for the most opioid dispensing, the opioid dispensing rate per 100 000 people declined from 301.7 to 184.9 MMEs (−38.7%) and mean total MMEs per prescription declined from 495.3 to 274.7 MMEs (−44.5%).

**Table.** Rate and Dosing of Opioid Prescriptions From US Surgeons

Specialty	Opioid dispensing rate (% of total across specialties)^a^			Mean total MMEs/prescription			MMEs per 100 000 people (% of total across specialties)		
	January 2016	December 2022	% Change	January 2016	December 2022	% Change	January 2016	December 2022	% Change
Cardiothoracic surgery	0.09 (0.01)	0.04 (0.01)	−49.6	511.0	146.0	−71.4	44 (0.02)	6 (0.01)	−85.6
General surgery (including colorectal and transplant)	100.7 (15.2)	77.4 (18.2)	−23.2	345.8	175.1	−49.4	34 841 (12.7)	13 555 (14.3)	−61.1
Neurosurgery	29.8 (4.5)	13.9 (3.3)	−53.2	831.2	468.7	−43.6	24 766 (9.0)	6535 (6.9)	−73.6
Obstetrics and gynecology	103.9 (15.7)	59.3 (13.9)	−42.9	274.9	156.2	−43.2	28 561 (10.4)	9262 (9.8)	−67.6
Ophthalmology	8.8 (1.3)	4.5 (1.1)	−48.6	230.6	89.3	−61.3	2021 (0.7)	402 (0.4)	−80.1
Orthopedic surgery (including hand surgery)	301.7 (45.6)	184.9 (43.4)	−38.7	495.3	274.7	−44.5	149 431 (54.6)	50 799 (53.7)	−66.0
Otolaryngology	29.9 (4.5)	26.7 (6.3)	−10.7	259.7	172.7	−33.5	7767 (2.8)	4612 (4.9)	−40.6
Pediatric surgery	3.6 (0.5)	1.7 (0.4)	−53.6	203.4	105.4	−48.2	737 (0.3)	177 (0.2)	−76.0
Plastic surgery	31.1 (4.7)	24.6 (5.8)	−20.9	355.4	187.2	−47.3	11 059 (4.0)	4606 (4.9)	−58.3
Thoracic surgery	5.5 (0.8)	2.0 (0.5)	−63.1	431.7	315.8	−26.9	2361 (0.9)	638 (0.7)	−73.0
Urology	39 (5.9)	26.9 (6.3)	−30.9	253.8	122.1	−51.9	9903 (3.6)	3291 (3.5)	−66.8
Vascular surgery	7.1 (1.1)	3.8 (0.9)	−45.9	317.6	172.6	−45.6	2257 (0.8)	664 (0.7)	−70.6
All specialties	661.2 (100)	426.0 (100)	−35.6	414.0	222.0	−46.4	273 746 (100)	94 548 (100)	−65.5

Abbreviation: MME, morphine milligram equivalent.

^a^ Opioid dispensing rate is defined as the number of dispensed opioid prescriptions per 100 000 people.

## Discussion

This cross-sectional study found that during 2016 to 2022, the rate and size of opioid prescriptions from US surgeons declined, but these declines were slower after mid-2020 compared with before 2020. During the initial months of the COVID-19 pandemic, the opioid dispensing rate declined, potentially owing to decreased surgical volume, while opioid prescription size increased, potentially because surgeons wrote larger discharge prescriptions owing to barriers to obtaining refills. However, these changes were transient.

Study limitations include lack of information on the specialty of advanced practice clinicians. Because of this limitation, surgical opioid prescriptions from these clinicians could not be identified or included in analyses.^6^

Despite large reductions in opioid prescribing, surgical opioid stewardship initiatives remain important. For example, the mean size of opioid prescription from surgeons was 44 pills in December 2022, more than patients typically need.^3^ Going forward, surgical opioid prescribing guidelines based on patient-reported opioid consumption could align prescribing with patient need.^3^

## Notes

Supplement 1.
eMethods.

Supplement 2.
Data Sharing Statement
