# Supplementary material for: Opioid Prescribing by US Surgeons, 2016-2022
Source: JAMA Netw Open. 2023 Dec 7;6(12):e2346426. doi: 10.1001/jamanetworkopen.2023.46426 (PMC10704275; doi:10.1001/jamanetworkopen.2023.46426)
Supplement: Supplement 1. — eMethods. [file jamanetwopen-e2346426-s001.pdf]

Supplemental Online Content

Zhang J, Waljee JF, Nguyen TD, et al. Opioid prescribing by US surgeons, 2016-2022. *JAMA Netw Open*. 2023;6(12):e2346426. doi:10.1001/jamanetworkopen.2023.46426

eMethods.

This supplemental material has been provided by the authors to give readers additional information about their work.

© 2023 Zhang J et al. *JAMA Network Open.*

eMethods. Details on methods

Population denominators

We derived estimates of the U.S. resident population from 2016-2022 using data from the Census Bureau. The population denominators from 2016-2019 were based on the 2010 Census, which may have underestimated population size compared with estimates from the 2020 Census. To account for this, we multiplied population denominators from 2016-2019 by a factor of 1.004921751.

**Year**	**Annual population denominator**
2016	324,530,748
2017	326,585,037
2018	328,295,376
2019	329,855,036
2020	331,501,080
2021	331,893,745
2022	332,838,183

Statistical analysis

Using joinpoint software from the National Cancer Institute, we fitted joinpoint regression models to assess for slope changes in the surgical opioid dispensing rate and monthly total MMEs per prescription. We fitted models with 1-3 joinpoints and selected the model with the best fit using permutation tests. Total MMEs were calculated by multiplying strength per dose, quantity, and MME conversion factors published by the Centers for Disease Control and Prevention.

© 2023 Zhang J et al. *JAMA Network Open.*
