# Supplementary material for: Opioid Prescribing by US Surgeons, 2016-2022
Source: JAMA Netw Open. 2023 Dec 7;6(12):e2346426. doi: 10.1001/jamanetworkopen.2023.46426 (PMC10704275; doi:10.1001/jamanetworkopen.2023.46426)
Supplement: Supplement 2. — Data Sharing Statement [file jamanetwopen-e2346426-s002.pdf]

Data Sharing Statement

Zhang. Opioid Prescribing by US Surgeons, 2016-2022. *JAMA Netw Open*. Published online December 7, 2023. doi:10.1001/jamanetworkopen.2023.46426

Data
Data available: No

Additional Information
Explanation for why data not available: The data used in this study is proprietary, so we are unable to make it available.
